# A screen for kinase inhibitors identifies antimicrobial imidazopyridine aminofurazans as specific inhibitors of the *Listeria monocytogenes* PASTA kinase PrkA

**DOI:** 10.1074/jbc.M117.808600

**Published:** 2017-08-16

**Authors:** Adam J. Schaenzer, Nathan Wlodarchak, David H. Drewry, William J. Zuercher, Warren E. Rose, Rob Striker, John-Demian Sauer

**Affiliations:** From the Departments of ‡ Medical Microbiology and Immunology and; § Medicine, University of Wisconsin-Madison, Madison, Wisconsin 53706,; the ¶ Structural Genomics Consortium-University of North Carolina at Chapel Hill (SGC-UNC), University of North Carolina at Chapel Hill Eshelman School of Pharmacy, University of North Carolina at Chapel Hill, Chapel Hill, North Carolina 27599,; the ‖ School of Pharmacy, University of Wisconsin-Madison, Madison, Wisconsin 53705, and; the ** Department of Molecular and Cell Biology, W. S. Middleton Memorial Veteran's Hospital, Madison, Wisconsin 53705

**Keywords:** antibiotics, bacterial protein kinase, bacterial signal transduction, drug discovery, drug screening, Listeria monocytogenes, PASTA Kinase, aminofurazans, β-lactams

## Abstract

Bacterial signaling systems such as protein kinases and quorum sensing have become increasingly attractive targets for the development of novel antimicrobial agents in a time of rising antibiotic resistance. The family of bacterial Penicillin-binding-protein And Serine/Threonine kinase-Associated (PASTA) kinases is of particular interest due to the role of these kinases in regulating resistance to β-lactam antibiotics. As such, small-molecule kinase inhibitors that target PASTA kinases may prove beneficial as treatments adjunctive to β-lactam therapy. Despite this interest, only limited progress has been made in identifying functional inhibitors of the PASTA kinases that have both activity against the intact microbe and high kinase specificity. Here, we report the results of a small-molecule screen that identified GSK690693, an imidazopyridine aminofurazan-type kinase inhibitor that increases the sensitivity of the intracellular pathogen *Listeria monocytogenes* to various β-lactams by inhibiting the PASTA kinase PrkA. GSK690693 potently inhibited PrkA kinase activity biochemically and exhibited significant selectivity for PrkA relative to the *Staphylococcus aureus* PASTA kinase Stk1. Furthermore, other imidazopyridine aminofurazans could effectively inhibit PrkA and potentiate β-lactam antibiotic activity to varying degrees. The presence of the 2-methyl-3-butyn-2-ol (alkynol) moiety was important for both biochemical and antimicrobial activity. Finally, mutagenesis studies demonstrated residues in the back pocket of the active site are important for GSK690693 selectivity. These data suggest that targeted screens can successfully identify PASTA kinase inhibitors with both biochemical and antimicrobial specificity. Moreover, the imidazopyridine aminofurazans represent a family of PASTA kinase inhibitors that have the potential to be optimized for selective PASTA kinase inhibition.

## Introduction

Antibiotics, in particular the β-lactams, are considered one of the greatest medical advances of the 20th century since their discovery and widespread use in the 1940's (1). However, due to the misuse of these life-saving drugs, antibiotic-resistant strains of bacteria such as carbapenem-resistant Enterobacteriaceae (CRE), vancomycin-resistant *Enterococcus* (VRE), and methicillin-resistant *Staphylococcus aureus* (MRSA) are emerging at an alarming rate (2, 3). The rapid evolution of resistance to available antibiotics currently outpaces the rate of development of new, effective treatments and highlights the need for the development of truly novel antimicrobial strategies (4, 5). One new strategy is the pursuit of novel compounds that target microbial signaling cascades that are relatively overlooked by traditional methods of antibiotic development. Reversible protein phosphorylation by bacterial kinases is one such process that has been garnering attention within the past decade as a potential target for truly novel antibiotics (6, 7).

Prokaryotic protein phosphorylation was originally thought to occur predominantly on histidine and aspartate residues phosphorylated by two-component systems in a fashion distinct from eukaryotic kinases (8, 9). However, since the discovery of *pkn1*, a bacterial serine/threonine kinase with high structural homology to eukaryotic protein kinases (10), genomic studies have shown eukaryotic-like serine/threonine kinases (eSTKs)^3^ to be near ubiquitous in bacteria (11). Specifically, many important Gram-positive pathogens have transmembrane eSTKs known as Penicillin-binding-protein And Ser/Thr kinase-Associated (PASTA) kinases (12). In a variety of different pathogens, PASTA kinases have been found to regulate cell wall homeostasis (13–17), germination (18, 19), metabolism (20, 21), biofilm formation (22), and virulence (23–32). The PASTA kinase PknB is essential in *Mycobacterium tuberculosis* (26, 33), whereas genetic deletion of homologs in other species has been linked to increased susceptibility to β-lactam antibiotics (13, 24, 25, 34). These phenotypes have led to interest in PASTA kinases as potential antibiotic targets in pathogens ranging from *M. tuberculosis* and *S. aureus* to *Listeria monocytogenes*. As a proof of principle, we previously demonstrated that pharmacologic inhibition of the PASTA kinase PrkA by the nonspecific kinase inhibitor staurosporine increases the susceptibility of the intracellular pathogen *L. monocytogenes* to β-lactams in broth culture (34); however, staurosporine's high promiscuity among eukaryotic kinases makes it remarkably toxic and undermines its usefulness as a candidate for therapeutic development (35). Staurosporine's hallmark toxicity highlights the necessity for kinase inhibitors that are selective for a limited number of targets.

Extensive efforts have been put forth to probe the biochemistry of eukaryotic kinases and identify structural features that can be exploited by selective kinase inhibitors for the treatment of a variety of human diseases, most notably cancer (36). Such a wealth of established knowledge can be harnessed to probe bacterial kinase biochemistry and engineer inhibitors that act as selective antibiotics. Furthermore, the abundance of available small molecule kinase inhibitor libraries can be mined for bacterial kinase-selective scaffolds. Here, we report that GSK690693, an imidazopyridine aminofurazan (IPA) identified in a small molecule kinase inhibitor library, sensitizes *L. monocytogenes* to various β-lactams. We show that other members of the IPA family inhibit PrkA biochemically and sensitize *L. monocytogenes* to β-lactams to varying degrees. Finally, we demonstrate selectivity for the *L. monocytogenes* PASTA kinase both at the biochemical and microbiological level as compared with the *S. aureus* PASTA kinase Stk1 on an amino acid level. Taken together, our data validate the potential to exploit PASTA kinases as druggable targets and establish GSK690693 and other IPAs as both lead compounds and valuable tools to investigate PASTA kinase biology.

## Results

### GSK690693 sensitizes Listeria to β-lactam antibiotics

**Figure 1.**
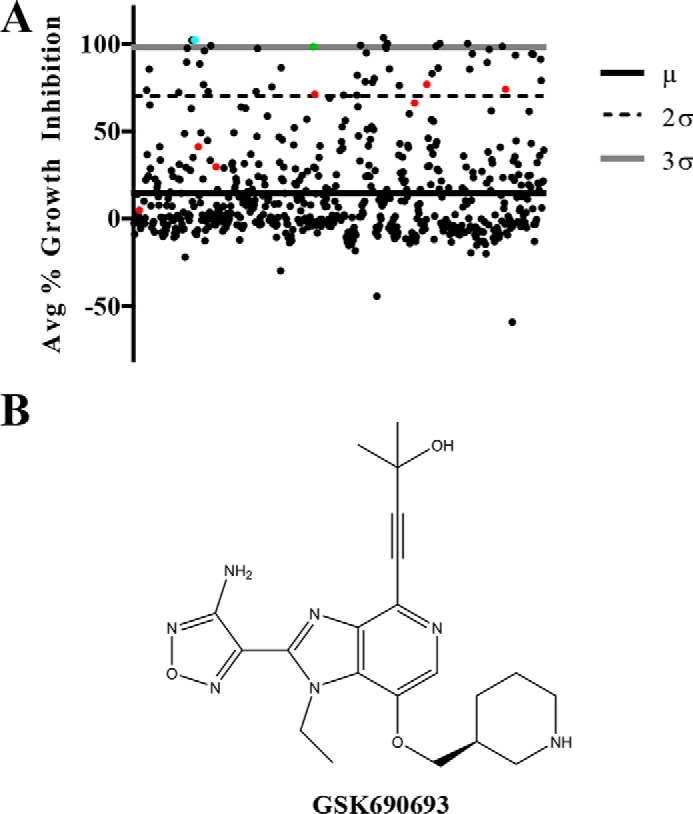
**Library screen identifies GSK690693 as a compound that sensitizes *L. monocytogenes* to ceftriaxone.** *A,* scatter plot representing percent growth inhibition of WT *L. monocytogenes* in the presence of a combination of a sublethal dose (1 μg/ml) of the β-lactam ceftriaxone and each compound in the screen. The *solid black line* represents the library mean (μ), and the *dashed black line* and *gray line* represent two (2σ) and three (3σ) S.D. above the library mean, respectively. The *cyan*, *green*, and *red data* points represent staurosporine, GSK690693, and other compounds from the IPA family, respectively. *B,* skeletal structure of GSK690693.

In a wide variety of important Gram-positive pathogens, PASTA kinases are essential for resistance to β-lactam antibiotics (13, 25, 34). We have previously demonstrated that either genetic deletion or pharmacologic inhibition of the PASTA kinase PrkA with staurosporine sensitizes *L. monocytogenes* to β-lactams (34). To identify specific (and therefore potentially less toxic) inhibitors of PrkA, we screened 625 small molecule kinase inhibitors from the GlaxoSmithKline Published Kinase Inhibitor Set (PKIS) (37, 38) and Selleck kinase inhibitor libraries against wild-type *L. monocytogenes* strain 10403s in the presence of a sublethal dose of the β-lactam ceftriaxone (Fig. 1*A*). Sixteen compounds potentiated inhibition of *L. monocytogenes* growth by ceftriaxone at 3 S.D. or more above the mean including the positive control staurosporine (Fig. 1*A*, *blue dot*). Of these, nine compounds failed to show a dose-response, and an additional three showed β-lactam independence in secondary screens. The four remaining validated compounds were LY2228820, GSK690693, and our previously published compounds staurosporine and AZD5438 (supplemental Fig. S1). We selected GSK690693 (Fig. 1, *A*, *green dot,* and *B*) for further analysis due to the presence of structural congeners in the screen (Fig. 1*A*, *red dots*).

**Figure 2.**
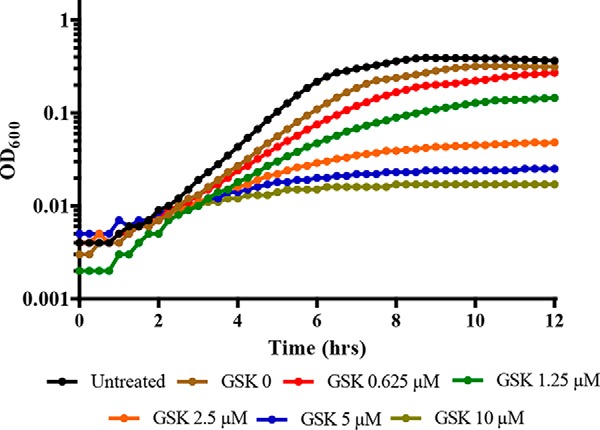
**GSK690693 potentiates the inhibitory action of ceftriaxone in a dose-dependent manner.** Growth curves of WT *L. monocytogenes* grown in the presence of 2.5 μg/ml of ceftriaxone and increasing concentrations (μm) of GSK690693. *Curves* are representative of 3 independent trials.

Based on our previous investigation of staurosporine, we hypothesized that GSK690693 would also sensitize *L. monocytogenes* to other β-lactam antibiotics. To test this hypothesis, we determined the minimal inhibitory concentration (MIC) values of various antibiotics against wild-type *L. monocytogenes* in the presence and absence of 20 μm GSK690693 (Table 1). Importantly, GSK690693 sensitized *L. monocytogenes* to other β-lactams as well, including ceftriaxone, ampicillin, and meropenem, but had no β-lactam-independent effects up to 20 μm (supplemental Fig. S2), consistent with the lack of β-lactam-independent growth defects in Δ*prkA* mutants (34). Although GSK690693 had no effect on the MIC values of the non-β-lactams vancomycin or kanamycin, it did sensitize *L. monocytogenes* to the TarO inhibitor tunicamycin, consistent with the role of PASTA kinases in resistance to tunicamycin (23, 39). To confirm that GSK690693 potentiation of β-lactam and tunicamycin sensitivity was through PASTA kinase inhibition, we tested the sensitivity of the Δ*prkA* mutant to the same antibiotics in the presence and absence of 20 μm GSK690693. GSK690693 afforded the Δ*prkA* strain no additional sensitization to any antibiotic tested, suggesting that the activity of GSK690693 is PrkA kinase-dependent (Table 1). Finally, GSK690693 sensitized wild-type *L. monocytogenes* in a concentration-dependent manner to a fixed subinhibitory concentration of ceftriaxone, demonstrating the reciprocal dose dependence of the kinase inhibitor-β-lactam potentiation (Fig. 2). Taken together, our data demonstrates that GSK690693 sensitizes *L. monocytogenes* to β-lactam antibiotics through PrkA inhibition.

**Table 1:** **MIC of various antibiotics against WT and Δ*prkA* strains +/− 20 μm GSK690693** Data presented as median of at least three biological replicates with the range in parentheses.

GSK690693	MIC (μg/ml)			
	10403s		Δ*prkA*	
	−	+	−	+
Ceftriaxone	8 (4, 8)	1 (0.5, 1)	0.0625 (0.03125, 0.25)	0.0625 (0.03125, 0.25)
Ampicillin	0.25 (0.25, 0.25)	0.0625 (0.0625, 0.125)	0.03125 (0.03125, 0.03125)	0.03125 (0.03125, 0.03125)
Meropenem	0.25 (0.25, 0.25)	0.125 (0.125, 0.25)	0.03125 (0.03125, 0.0625)	0.0625 (0.0625, 0.0625)
Tunicamycin	32 (32, 32)	0.5 (0.5, 1)	0.5 (0.5, 1)	0.5 (0.5, 1)
Vancomycin	2 (2, 4)	2 (2, 4)	1 (1, 1)	1 (1, 1)
Kanamycin	8 (8, 8)	8 (8, 8)	8 (8, 8)	8 (8, 8)

### GSK690693 inhibits PrkA in vitro

**Figure 3.**
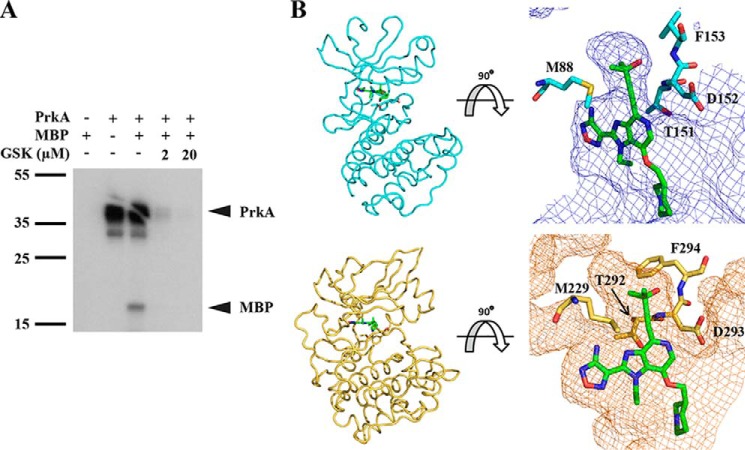
**GSK690693 inhibits the PrkA kinase domain *in vitro*.** *A*, autoradiography blot of purified PrkA kinase domain from *L. monocytogenes* and the nonspecific phosphoacceptor substrate MBP in the presence or absence of GSK690693. Blot is representative of 3 independent trials. *B* (*top*), GSK690693 docked *in silico* into the threaded model of the kinase domain of PrkA (*bottom*) crystal structure of GSK690693 bound to human AKT (PDB ID 3d0e). Gatekeeper methionine and xDF residues are represented as *sticks*.

To assess the basis of kinase inhibitor/β-lactam potentiation at the biochemical level and to begin to understand biochemical determinants of activity, we assessed the kinase activity of the purified kinase domain of PrkA in the presence of increasing concentrations of GSK690693. Analysis by autoradiography revealed that GSK690693 robustly inhibited both PrkA autophosphorylation and phosphorylation of the nonspecific phosphoacceptor myelin basic protein (MBP) at concentrations as low as 2 μm (Fig. 3*A*). Consistent with this finding, analysis of kinase inhibition in the Kinase-Glo® assay (Promega) resulted in a calculated IC_50_ value of 0.84 μm (Table 2). Finally, *in silico* docking of GSK690693 in the kinase domain of PrkA predicted the 2-methyl-3-butyn-2-ol (alkynol) moiety penetrating into the gatekeeper-guarded back pocket of the kinase active site, similar to the orientation and mechanism of inhibition previously reported for GSK690693 bound to the eukaryotic kinase AKT (Fig. 3*B*) (40). Taken together, our data demonstrate that GSK690693 can directly inhibit PrkA activity *in vitro*.

**Table 2:** **Summary of biochemical and microbiology data for various IPAs against *L. monocytogenes***

Compound	Relative IC_50_ (95% CI)	Ceftriaxone MIC
	μ*m*	μ*g/ml*
DMSO	NA*^a^*	8
GSK690693	0.84 (0.34, 2.00)	1
GSK554170A	0.45 (0.080, 2.49)	2
GSK614526A	0.42 (0.010, 17.17)	0.25
GSK902056A	0.41 (0.10, 1.63)	1
SB-747651A	NA	8

*^a^* NA, not applicable.

### Various IPAs display biochemical and microbiologic activity

**Figure 4.**
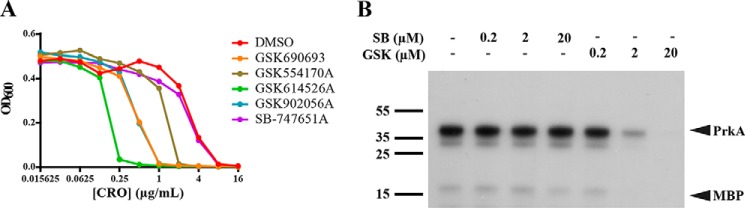
**IPAs potentiate ceftriaxone activity to varying degrees.** *A,* dose-response curves of *L. monocytogenes* growth *versus* ceftriaxone in the presence and absence of 10 μm IPAs. Curves are representative of 3 independent trials. *B,* autoradiography blot of purified PrkA kinase domain and MBP in the presence or absence of GSK690693 or SB-747651A. The blot is representative of 3 independent trials.

There were seven other members of the IPA family in our library screen (Fig. 1, *red dots*), three of which demonstrated potentiation with ceftriaxone at >2 S.D. above the mean of the screen and whose structures are shown in Table 3. To determine whether the IPAs represent a broadly applicable scaffold for PASTA kinase inhibitors we tested 3 additional compounds for biochemical activity and ceftriaxone potentiation: GSK554170A, GSK614526A, and GSK902056A (Fig. 4*A*, Table 2). All three compounds showed statistically similar biochemical activity to GSK690693; however, GSK554170A was 2-fold less potent than GSK690693 microbiologically, whereas GSK614526A was 4-fold more potent than GSK690693. Taken together, our data suggest that these variations in the R2 and R3 positions of the PIA scaffold may play a role in target accessibility and/or stability in the bacterial cytosol. In contrast, SB-747651A, an IPA that lacks the alkynol moiety, neither potentiated β-lactam sensitivity (Fig. 4*A*) nor inhibited biochemical activity (Fig. 4*B*). This is consistent with molecular modeling that implicates the alkynol moiety in stabilization of binding and kinase inhibition (Fig. 3*B*). Taken together, our findings demonstrate that multiple compounds of the IPA family are capable of augmenting ceftriaxone activity to varying degrees and further suggest that the IPA pharmacophore can be further optimized for β-lactam sensitization.

**Table 3:**
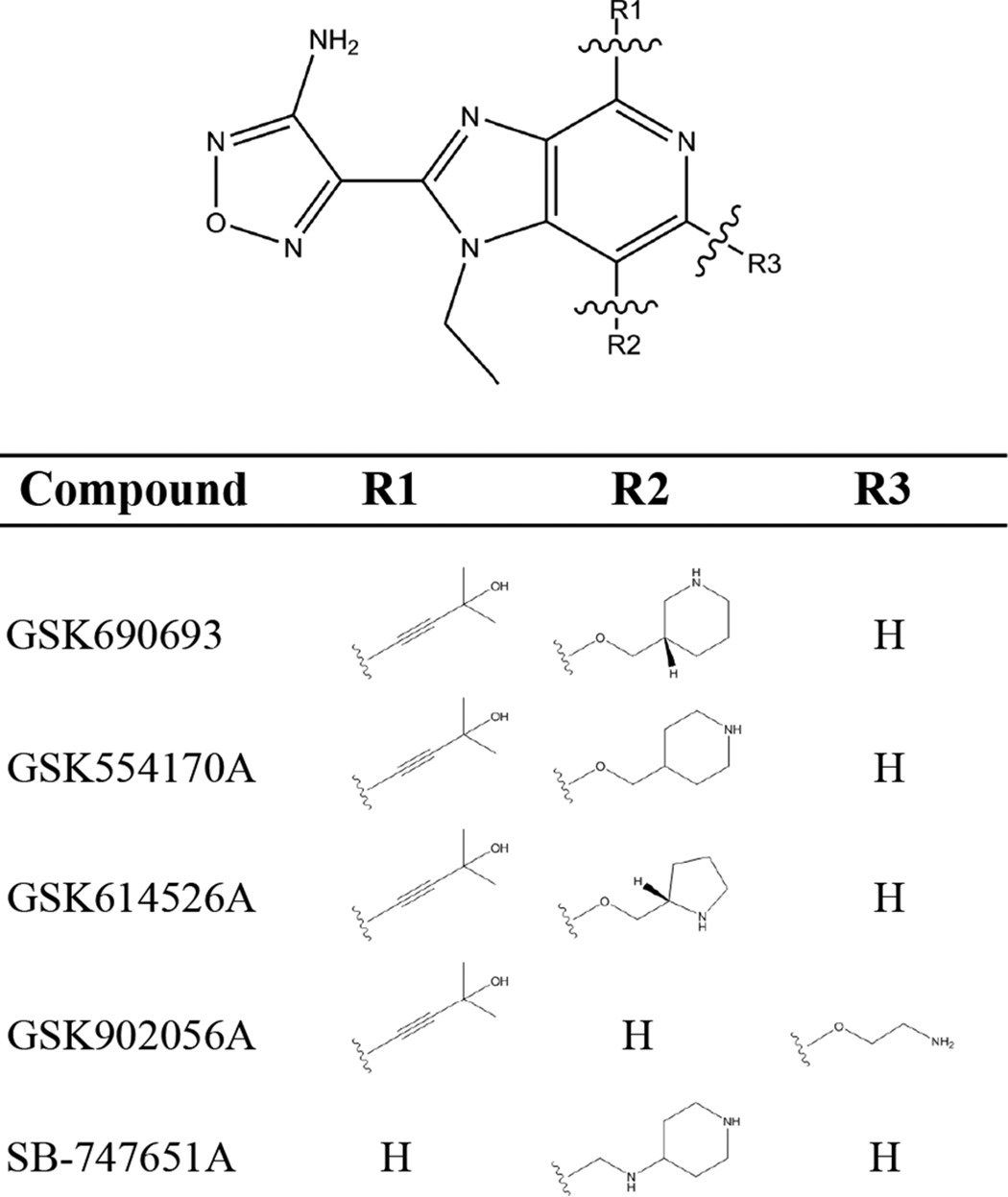
**Structures of various IPA family compounds**

### GSK690693 displays selectivity for PrkA over Stk1

**Figure 5.**
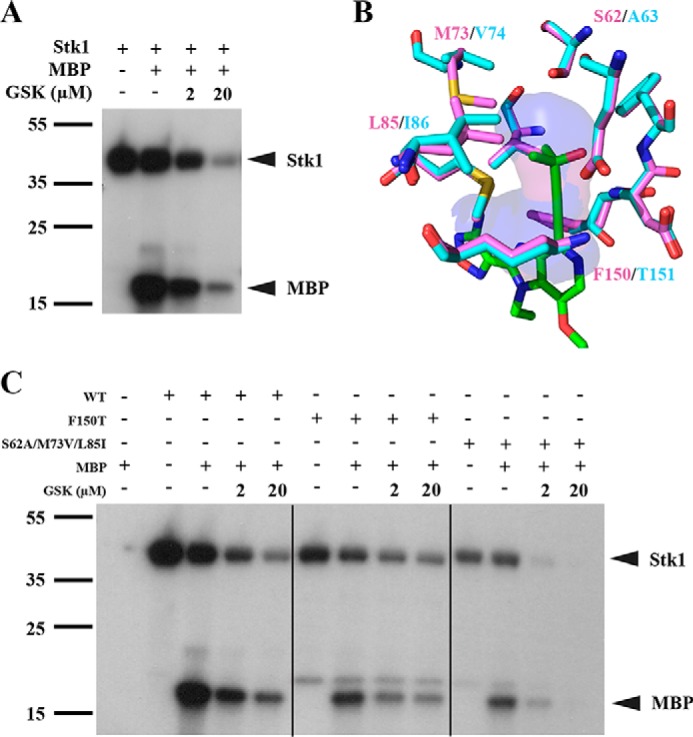
**Residues of the back pocket play a role in GSK690693 selectivity.** *A,* autoradiography blot of purified Stk1 kinase domain from *S. aureus* and MBP in the presence or absence of GSK690693. *B,* stick figure representation of the amino acids that constitute the back pocket of the PrkA (*cyan*) and Stk1 (*violet*) kinase domains. GSK690693 (*green sticks*) is docked into the back pocket, which is represented by the translucent cavity surface. *C*, autoradiography blot of purified WT Stk1, F150T mutant, S62A/M73V/L85I triple mutant, and MBP in the presence or absence of GSK690693. Blots are representative of 3 independent trials.

As PASTA kinases are highly conserved in a variety of important human pathogens, we hypothesized that GSK690693 may act on other PASTA kinases as well. Surprisingly, we observed that GSK690693 was significantly less potent against the purified kinase domain of Stk1, the PASTA kinase homolog from *Staphylococcus aureus* (Fig. 5*A*), with a predicted IC_50_ greater than 40 μm by a Kinase-Glo® assay (supplemental Fig. S3*E*). Consistent with this finding, neither the ceftriaxone nor oxacillin MIC for the methicillin-resistant *S. aureus* strain LAC was altered in the presence of GSK690693, although there was a reproducibly minor slowdown in growth in the presence of GSK690693 relative to antibiotics alone (supplemental Fig. S3, *D* and *E*) consistent with the low level inhibition of Stk1 observed biochemically. In contrast, Δ*stk1* mutants are potently sensitized to both β-lactam antibiotics (Supplemental Fig. S3, *B* and *C*) as previously described (24). Importantly, Stk1 can be biochemically inhibited by the kinase inhibitor indirubin-3′-monoxime, which shows relative selectivity for Stk1 over PrkA (supplemental Fig. S4). Consistent with this, three recent papers have identified small molecule inhibitors of Stk1 that are structurally distinct from the IPAs we identified as PrkA inhibitors (41–43). These data suggest that GSK690693 selectively inhibits the kinase activity of PrkA relative to Stk1 and highlight the possibility of designing non-broad spectrum, pathogen-specific inhibitors.

Intrigued by the divergent activity against the two kinase domains, we utilized *in silico* modeling to identify potential structural deviations that might explain the selectivity. Examination of the back pockets of the Stk1 and PrkA kinase domains revealed high sequence conservation with only four divergent residues: Ser^62^(Stk1)/Ala^63^(PrkA), Met^73^/Val^74^, Leu^85^/Ile^86^, and Phe^150^/Thr^151^ (Fig. 5*B*). As Phe^150^ and Thr^151^ sit at the entrances of their respective kinase's back pocket just upstream of the catalytic DFG motif, we hypothesized that a bulky “xDFG” residue at this position (such as Phe^150^ in Stk1) may deny GSK690693's alkynol group access to the pocket, whereas a smaller residue (such as Thr^151^ of PrkA) may be permissible. However, when these residues are swapped between kinases, we found that PrkA T151F has greatly reduced intrinsic kinase activity (supplemental Fig. S5), whereas Stk1 F150T sensitivity to GSK690693 is unaffected (Fig. 5*C*). Alternatively, the three other divergent residues (Met^73^/Val^74^, Leu^85^/Ile^86^, and Ser^62^/Ala^63^) might alter the size and polarity of the pocket in a way that generates selectivity. Strikingly, an Stk1 S62A/M73V/L85I triple mutant is more sensitive to GSK690693 relative to the wild-type kinase domain (Fig. 5*C*), although there is also a minor decrease in intrinsic kinase activity. Overall, our data suggests that, at least in part, selectivity of GSK690693 for PrkA over Stk1 is not due to the xDFG residue but rather is mediated by the size and charge of the back pocket that stabilizes the alkynol moiety facilitating inhibition. Additional point mutants altering the character of the back pocket in addition to synthesizing alkynol modifications will help to establish a formal SAR and may instruct the rational design of species-specific kinase inhibitors in the future.

## Discussion

Eukaryotic kinases have been a target of the pharmaceutical industry for decades owing to their central role in a variety of cancers and other diseases (44, 45). As of 2015, 27 protein kinase inhibitors are FDA-approved for use in the clinic (46). In light of this relative success in eukaryotes, prokaryotic protein kinases have begun to be investigated as potentially novel antibiotic targets. A highly conserved family of bacterial kinases, the PASTA kinases, have high levels of conservation with eukaryotic kinases and play central roles in processes ranging from metabolism and basic bacterial physiology to regulation of virulence and β-lactam antibiotic susceptibility. As such, efforts are being put forth to identify small molecule inhibitors of the PASTA kinases (34, 42, 47, 48). As these efforts progress, it will be important to consider the need for a better understanding of the biochemistry of PASTA kinase inhibition to aid in the development of selective kinase inhibitors. Here, we present GSK690693 and other IPAs as novel inhibitors of the *L. monocytogenes* PASTA kinase PrkA and as a tool to better understand PASTA kinase biochemistry.

We identified 16 compounds, including GSK690693, which inhibited growth of *L. monocytogenes* in the presence of a sublethal dose of a β-lactam by performing a small (625 compound) primary screen of compounds known to possess a pharmacophore with kinase-inhibiting attributes. We chose GSK690693 above others due to its dependence on PrkA, its dependence on the presence of a β-lactam, and its dose dependence. Although several inhibitors with PrkA-specific activity may have been missed in our screen, the utilization of a microbiological screen rather than a biochemical screen immediately overcame a significant barrier that has been encountered in screens to identify *M. tuberculosis* PknB inhibitors, namely identification of compounds capable of entering into the bacterium (47).

Seven congeners of GSK690693 were also present in our screen, each possessing the characteristic alkynol moiety and IPA scaffold but varying in the position and molecular structures of their respective side chains. Those that we further characterized all had statistically similar IC_50_ values although their microbiologic activity varied. This suggests a varying ability of these compounds to access their target in the bacterial cytosol or a difference in stability once in the cytosol, further exemplifying the need to modify “Lipinski's rule of 5” to account for the bacterial cell wall when performing antibiotic development. Our data indicate that modifications to the R2 or R3 position may be more important in dictating target accessibility rather than biochemical activity.

GSK690693's eukaryotic SAR has established that the alkynol moiety penetrates into AKT's gatekeeper-guarded back pocket to stabilize binding (40). Interestingly, SB-747651A is completely unable to inhibit PrkA activity and sensitize *L. monocytogenes* to a β-lactam, likely due to the lack of the alkynol moiety on the IPA scaffold. Although effects of SB-747651A's R2 side chain cannot be ruled out, it is worth noting that all other tested IPAs that possess the alkynol moiety have some efficacy against *L. monocytogenes* PrkA, regardless of the side chain structure at R1 or R2.

Due to an expansion in our knowledge of the effects of current antibiotics on the human microbiome (50, 51), one of the challenges of antimicrobial development has become finding antimicrobial compounds that are selective for the pathogen of interest without disruption of normal microbiota or collateral resistance effects. Therefore, we were intrigued to find that GSK690693 showed selectivity for the PrkA kinase domain over Stk1, the PASTA kinase from *S. aureus* that shows 49% identity with PrkA across its kinase domain (Figs. 3*A* and 5*A*). PASTA kinase inhibitors with varying degrees of biochemical activity have been identified for *M. tuberculosis* PknB (19, 33, 47, 48, 52, 53), *Enterococcus faecalis* IreK (54), *Bacillus subtilis* PrkC (18), *L. monocytogenes* PrkA (34), and staphylococcal Stk1 (22, 41–43); however, this work is the first to investigate the selectivity of an inhibitor between two PASTA kinases. Given the importance of the gatekeeper-guarded back pocket in the eukaryotic SAR of GSK690693, we investigated differences in the back pockets of PrkA and Stk1. Mutation of the most obvious amino acid residue (Stk1 F150T) at the entrance to the pocket did not alter selectivity; however, mutation of the three divergent residues that contribute to the shape and depth of the back pocket profoundly affected the sensitivity of Stk1 to GSK690693 (Fig. 5*C*). To our knowledge, investigations in human kinases have not implicated these internal residues as contributing to selectivity (55, 56). GSK690693 has been well-established as relatively selective among eukaryotic kinases for isoforms of AKT (38, 40, 57), although this is the first time activity against specific bacterial kinases has been shown. Overall, GSK690693's selectivity between two highly similar kinases found in *L. monocytogenes* and *S. aureus* extends the concept that pathogen-specific inhibitors could be identified and developed (34).

The mechanism by which PrkA mediates tunicamycin resistance remains unknown. At low (non-lethal) concentrations, tunicamycin inhibits the activity of TarO, the enzyme required for the transfer of GlcNAc-1-phosphate from UDP-GlcNAc to undecaprenyl phosphate during wall teichoic acid synthesis (58). However, at higher (lethal) concentrations, tunicamycin also blocks the activity of MraY, the essential enzyme required for the transfer of phospho-MurNAc-pentapeptide to undecaprenyl phosphate during peptidoglycan synthesis (59). Sensitization to tunicamycin through PrkA inhibition may be due to PrkA-mediated regulation of one or both of these proteins directly as substrates. Alternatively, PrkA may act on the pathways further upstream or downstream of these proteins. Additionally, it is unknown why treatment with GSK690693 can achieve the maximum-expected sensitivity to tunicamycin (*i.e.* match the phenotype of a Δ*prkA* mutant) but cannot do the same for the β-lactams. It is possible that the tunicamycin phenotype is dependent solely on kinase activity, whereas the β-lactam phenotype might be dependent on both kinase activity and other non-enzymatic roles of PrkA. If true, then inhibition of kinase activity by GSK690693 would only be able to achieve a fraction of the phenotype of the genetic deletion. Determining if there are kinase activity-independent functions of PrkA is an active area of investigation.

As with tunicamycin, the exact mechanism by which PrkA mediates β-lactam resistance is not well-understood. A considerable number of enzymes and proteins involved in cell wall metabolism are directly phosphorylated by eSTKs in a variety of species, with examples including MurC (60), DivIVA/Wag31 (62), GpsB (14), and VraR (27). Such a span of substrates leads to the conclusion that PrkA (and the PASTA kinases in general) may play a role in many aspects of cell wall metabolism such as muropeptide synthesis, PBP function and localization, and the orchestration of cell elongation and septation. These possibilities are still under investigation.

In conclusion, we have identified GSK690693 and other IPAs as novel inhibitors of the PASTA kinase PrkA with the potential for increased selectivity among PASTA kinases. We have shown that GSK690693 potentiates β-lactam activity against *Listeria* in broth culture. Furthermore, we have begun to establish an SAR by demonstrating that the alkynol group and nature of the back pocket in which it is predicted to be bound is potentially important for PASTA kinase inhibition by IPAs. These studies represent a stepping stone in the development of new and selective antibiotic therapies that could breathe new life into an exhausted antibiotic class.

## Materials and methods

### Bacterial strains and growth conditions

All bacterial strains used in this study are listed in supplemental Table S1. Conditional deletion of *prkA* (Δ*prkA*) was achieved as previously described (34). All *L. monocytogenes* strains were grown in brain-heart infusion (BHI) medium at 30 °C in stationary overnight until the strains reached stationary phase. Cultures were then back-diluted 1:50 for *in vitro* growth experiments. All *S. aureus* strains were grown in tryptic soy broth medium at 37 °C shaking overnight until stationary phase. Cultures were then back-diluted to an *A*_600_ of 0.06 for *in vitro* growth experiments. *Escherichia coli* strains XL-1Blue and Rosetta BL21 were used for subcloning and protein expression, respectively. When needed, chloramphenicol (Sigma) was used at 10 μg/ml and carbenicillin (Sigma) was used at 100 μg/ml. For all broth growth assays, GSK690693 (Selleck Chemicals, Houston, TX) was used at a final concentration of 20 μm (2% DMSO) unless otherwise specified in the figure legends.

### Library screen

The PKIS1 and Selleck libraries were obtained via the University of Wisconsin Carbone Cancer Center's Small Molecule Screening Facility. Overnight cultures were back-diluted 1:50 into fresh BHI medium containing 1 μg/ml of ceftriaxone and either library compounds (final concentration: 10 μm in 2% DMSO) or DMSO (final concentration: 2%). Growth was measured as an optical density at 600 nm (*A*_600_) in 15-min intervals for 12 h in a 96-well format using an Eon microplate spectrophotometer or Synergy HT microplate spectrophotometer (BioTek Instruments, Inc., Winooski, VT) (growth conditions: 37 °C, linear shaking). Each compound was screened twice. Percent inhibition was calculated as (1 − (OD*_x_*/OD_CRO_)) × 100, where OD*_X_* is the end point *A*_600_ for a culture treated with both ceftriaxone and compound X, and OD_CRO_ is the end point *A*_600_ for a culture treated with ceftriaxone alone. Compounds that inhibited growth 3 S.D. greater than the library mean were further verified for dose responsiveness and β-lactam dependence.

### MIC determination

Overnight cultures of *L. monocytogenes* were back-diluted 1:50 into fresh BHI medium containing 2-fold dilutions of the antibiotics ampicillin, ceftriaxone, meropenem, tunicamycin, kanamycin, or vancomycin in the presence or absence of GSK690693. *A*_600_ was measured to monitor growth of the microdilutions as described above. *S. aureus* overnight cultures were back-diluted to an *A*_600_ of 0.06 into cation-adjusted Mueller-Hinton medium containing 2-fold dilutions of the antibiotic ceftriaxone in the presence or absence of GSK690693. *A*_600_ was measured to monitor growth of the microdilutions for 16 h. MICs were defined as the lowest concentration of antibiotic required to prevent turbidity in broth visible by eye. Each MIC experiment was performed at least three times.

### Kinase domain protein expression and purification

The *prkA* and *stk1* kinase domains (1–338 and 1–348, respectively) were subcloned into the expression vector pGEX-2T as previously described (34). The plasmids were transformed into *E. coli* Rosetta BL21 cells, and protein expression was verified by SDS-PAGE. The bacteria were pelleted by centrifugation and resuspended in lysis buffer (25 mm Tris, pH 8.0, 150 mm NaCl, 1 mm dithiothreitol, 10 mm MgCl_2_) containing 2 μg/ml of DNase, 2 μg/ml of aprotinin, 1 μg/ml of leupeptin, and 25 μg/ml of phenylmethylsulfonyl fluoride (PMSF). Cells were lysed by sonication, and cell debris was pelleted by centrifugation for 20 min. Supernatant was passed through GS4FF affinity resin columns at 4 °C; columns were then rinsed with lysis buffer, and the protein eluted with elution buffer (50 mm Tris, pH 8.0, 5 mm NaCl, 3 mm DTT, 20 mm reduced glutathione, 1 mm MgCl_2_). Eluted protein was then digested overnight at 4 °C with 1/20 (w/w) thrombin. Digested protein was passed through a HiPrep Q16 10FF anionic exchange column (Buffer A: 20 mm Tris, pH 8.0, 1 mm DTT) via an ÄKTA purifier (GE Healthcare Life Sciences); protein was eluted off the column with a 0–50% gradient of Buffer B (20 mm Tris, pH 8.0, 1 mm DTT, 1 m NaCl). Target fractions were then combined and passed through GS4FF affinity columns as described above. The flow-through was concentrated via spin columns and passed through a Sephadex 75 size exclusion column on the ÄKTA purifier (Running Buffer: 10 mm Tris, pH 8.0, 150 mm NaCl, 1 mm DTT, 10 mm MgCl_2_). Fractions were tested for purity by SDS-PAGE and combined. For Stk1 purification, MgCl_2_ was replaced with MnCl_2_ throughout.

### Generation of kinase mutants

All plasmids generated in this study are listed in supplementary Table S2. To generate the Stk1-F150T mutant, plasmid pGEX-2T-Stk1 was digested with BamHI and KpnI (New England Biolabs) to remove the wild-type N-terminal Stk1 sequence. A gBlock gene fragment (Integrated DNA Technologies) consisting of the excised N-terminal sequence with a F150T (*T1404A, T1405C*) mutation was ligated into the digested plasmid to yield pGEX-2T-Stk1 F150T. To generate the PrkA T151F mutant, a similar process was performed on the pGEX-2T-PrkA plasmid, utilizing BamHI and EcoRI restriction sites and a gBlock gene fragment with a T151F (*A451T, C452T, A453T*) mutation to yield pGEX-2T-PrkA T151F. Finally, to generate the Stk1 S62A/M73V/L85I triple mutant, a similar process was performed on the pGEX-2T-Stk1 plasmid, utilizing BamHI and KpnI restriction sites and a gBlock gene fragment with S62A (*T187A*), M73V (*A217G, G219T*), and L85I (*T253A, A255T*) mutations to yield pGEX-2T-Stk1 S62A/M73V/L85I. All mutant constructs were validated by sequencing. Mutant constructs were transformed into *E. coli* Rosetta BL21 cells and protein expression and purification were performed in the same fashion as the wild-type constructs.

### Generation of Δstk1 mutant

Regions 1000 bp in size directly upstream and downstream were amplified with primer pairs JDS88/BK34 and BK35/JDS89, respectively (supplemental Table S3). These regions were fused by “splice by overlap” PCR and ligated into the plasmid pJB38 utilizing the SacI and XmaI restriction sites to yield pJB38-Δstk1. The construct was electroporated into the *S. aureus* strain RN4220 then transduced into LAC by phage transduction. Deletion of the *stk1* gene was then performed by pJB38-mediated allelic exchange as described by Bose *et al.* (63). Successful deletion of *stk1* was validated by PCR.

### Kinase-Glo® assay

The kinase assays were performed using the KinaseGlo® reagent from Promega. All reactions were done in 50 μl volume. The buffer used for all kinase assays was 10 mm Tris-HCl, pH 7.4, 150 mm NaCl, 1 mm DTT, and 1 mm MgCl_2_. Drugs in 5 mm DMSO were diluted in kinase buffer to 1/2 the final concentration using a serial dilution from 20 to 0 μm. The final DMSO concentration in the reactions was no more than 0.4%. PrkA(1–338) was added to the drugs to a final concentration of 2.0 μm and allowed to incubate for 10 min at 37 °C. ATP and MBP (Novatein Biosciences, Woburn, MA) were added for a final concentration of 100 and 40 μm, respectively, initializing the reaction. After a half-hour incubation, the reaction was stopped by the addition of 50 μl of KinaseGlo® reagent, and the signal was allowed to stabilize for 10 min at room temperature per the product manual. The plate was read using luminescence detection on a Synergy HT detector (BioTek) and the data were collected using the Gen5 2.0 software (BioTek). The data were transformed to log scale and non-linear regression was performed in PRISM using the variable slope 4-parameter model for enzyme inhibition to determine IC_50_.

### In vitro protein phosphorylation

2 μm kinase domain, 10 μm MBP (Novatein Biosciences), and various concentrations of kinase inhibitors were incubated on ice for 10 min, then added to a mixture of 10 mm Tris, pH 7.4, 150 mm NaCl, 1 mm MgCl_2_ (50 μm MnCl_2_ for Stk1 and its mutants), 50 μm ATP, and 1 μCi of [γ-^32^P]ATP. Reactions were incubated at 37 °C for 1 h and terminated by the addition of 6× SDS loading buffer. Samples were run on an SDS-PAGE gel then fixed for 2 h in fixation solution (40% methanol, 5% glycerol, 10% glacial acetic acid). Fixed gels were dried for 1 h and blots were visualized by autoradiography.

### In silico modeling

The primary sequences of the PrkA and Stk1 kinase domains (residues 1–270) were threaded onto the crystal structure of the kinase domain of PknB from *M. tuberculosis* (PDB ID 1O6Y) using the Phyre2 server's one-to-one threading (64). Hydrogen atoms and Gasteiger-Huckel charges were added, and energy minimization was performed using SYBYL-X1.2 (65). The structure of GSK690693 was downloaded as a MOL2 file from the ZINC database (66). Hydrogen atoms and Gasteiger-Huckel charges were added, and energy minimization was performed using SYBYL-X1.2. GSK690693 was docked into a 66 × 66 × 66 unit grid encompassing the kinases' active site clefts using the docking program Autodock's Lamarckian genetic algorithm (49). Models were visualized using PyMOL (61). The reference crystal structure of GSK690693 bound to human AKT (PDB ID 3d0e) was directly downloaded from the PDB and visualized in PyMOL without modifications.

## Author contributions

A. S. performed experiments, analyzed results, and wrote manuscript. N. W. performed experiments, wrote manuscript, and analyzed results. D. D., W. Z., W. E. R., R. S., and J. D. S. were involved in study design, data analysis, and manuscript preparation.

## Supplementary Material

Supplemental Data

## Footnotes

3 The abbreviations used are:

eSTK: eukaryotic-like serine/threonine kinase
PASTA: penicillin-binding-protein and Ser/Thr kinase-associated
IPA: imidazopyridine aminofurazan
MIC: minimal inhibitory concentration
MBP: myelin basic protein
BHI: brain-heart infusion
PDB: Protein Data Bank.

## References

[1] Lewis K. (2013) Platforms for antibiotic discovery. Nat. Rev. Drug Discov. 12, 371–387 23629505 10.1038/nrd3975

[2] Frieden T. (2013) Antibiotic resistance threats in the United States, 2013. CDC CS239559-B

[3] O'Neill J. (2014) Antimicrobial resistance: tackling a crisis for the health and wealth of nations. Review on Antimicrobial Resistance, HM Government and the Wellcome Trust.

[4] Spellberg B., Powers J. H., Brass E. P., Miller L. G., and Edwards J. E. (2004) Trends in antimicrobial drug development: implications for the future. Clin. Infect. Dis. 38, 1279–1286 15127341 10.1086/420937

[5] Projan S. J., and Shlaes D. M. (2004) Antibacterial drug discovery: is it all downhill from here? Clin. Microbiol. Infect. 10, 18–22 10.1111/j.1465-0691.2004.1006.x 15522036

[6] Kurosu M., and Begari E. (2010) Bacterial protein kinase inhibitors. Drug Dev. Res. 71, 168–187

[7] Pensinger D. A., Schaenzer A. J., and Sauer J. (2017) Do shoot the messenger: PASTA kinases as virulence determinants and antibiotic targets. Trends Microbiol. 10.1016/j.tim.2017.06.010 PMC5741517 28734616

[8] Stock J. B., Stock A. M., and Mottonen J. M. (1990) Signal transduction in bacteria. Nature 344, 395–400 2157156 10.1038/344395a0

[9] Hoch J. A. (2000) Two-component and phosphorelay signal transduction. Curr. Opin. Microbiol. 3, 165–170 10745001 10.1016/s1369-5274(00)00070-9

[10] Muñoz-Dorado J., Inouye S., and Inouye M. (1991) A gene encoding a protein serine/threonine kinase is required for normal development of *M. xanthus*, a Gram-negative bacterium. Cell 67, 995–1006 1835671 10.1016/0092-8674(91)90372-6

[11] Shi L., Potts M., and Kennelly P. J. (1998) The serine, threonine, and/or tyrosine-specific protein kinases and protein phosphatases of prokaryotic organisms: a family portrait. FEMS Microbiol. Rev. 22, 229–253 9862122 10.1111/j.1574-6976.1998.tb00369.x

[12] Yeats C., Finn R. D., and Bateman A. (2002) The PASTA domain: a β-lactam-binding domain. Trends Biochem. Sci. 27, 438 12217513 10.1016/s0968-0004(02)02164-3

[13] Beltramini A. M., Mukhopadhyay C. D., and Pancholi V. (2009) Modulation of cell wall structure and antimicrobial susceptibility by a *Staphylococcus aureus* eukaryote-like serine/threonine kinase and phosphatase. Infect. Immun. 77, 1406–1416 19188361 10.1128/IAI.01499-08 PMC2663143

[14] Foulquier E., Pompeo F., Freton C., Cordier B., Grangeasse C., and Galinier A. (2014) PrkC-mediated phosphorylation of overexpressed YvcK regulates PBP1 localization in *Bacillus subtilis mreB* mutant cells. J. Biol. Chem. 289, 23662–23669 25012659 10.1074/jbc.M114.562496 PMC4156092

[15] Fleurie A., Manuse S., Zhao C., Campo N., Cluzel C., Lavergne J. P., Freton C., Combet C., Guiral S., Soufi B., Macek B., Kuru E., VanNieuwenhze M. S., Brun Y. V., Di Guilmi A. M., Claverys J. P., Galinier A., and Grangeasse C. (2014) Interplay of the serine/threonine-kinase StkP and the paralogs DivIVA and GpsB in pneumococcal cell elongation and division. PLoS Genet. 10.1371/journal.pgen.1004275 PMC3983041 24722178

[16] Fridman M., Williams G. D., Muzamal U., Hunter H., Siu K. W., and Golemi-Kotra D. (2013) Two unique phosphorylation-driven signaling pathways crosstalk in *Staphylococcus aureus* to modulate the cell-wall charge: Stk1/Stp1 meets GraSR. Biochemistry 52, 7975–7986 24102310 10.1021/bi401177n

[17] Liebeke M., Meyer H., Donat S., Ohlsen K., and Lalk M. (2010) A metabolomic view of *Staphylococcus aureus* and its Ser/Thr kinase and phosphatase deletion mutants: involvement in cell wall biosynthesis. Chem. Biol. 17, 820–830 20797611 10.1016/j.chembiol.2010.06.012

[18] Shah I. M., Laaberki M.-H., Popham D. L., and Dworkin J. (2008) A eukaryotic-like Ser/Thr kinase signals bacteria to exit dormancy in response to peptidoglycan fragments. Cell 29, 997–1003 10.1016/j.cell.2008.08.039 PMC2892110 18984160

[19] Ortega C., Liao R., Anderson L. N., Rustad T., Ollodart A. R., Wright A. T., Sherman D. R., and Grundner C. (2014) *Mycobacterium tuberculosis* Ser/Thr protein kinase B mediates an oxygen-dependent replication switch. PLos Biol. 10.1371/journal.pbio.1001746 PMC3883633 24409094

[20] Leiba J., Hartmann T., Cluzel M. E., Cohen-Gonsaud M., Delolme F., Bischoff M., and Molle V. (2012) A novel mode of regulation of the *Staphylococcus aureus* catabolite control protein A (CcpA) mediated by Stk1 protein phosphorylation. J. Biol. Chem. 287, 43607–43619 23132867 10.1074/jbc.M112.418913 PMC3527947

[21] Lomas-Lopez R., Paracuellos P., Riberty M., Cozzone A. J., and Duclos B. (2007) Several enzymes of the central metabolism are phosphorylated in *Staphylococcus aureus*. FEMS Microbiol. Lett. 272, 35–42 17498211 10.1111/j.1574-6968.2007.00742.x

[22] Liu Q., Fan J., Niu C., Wang D., Wang J., Wang X., Villaruz A. E., Li M., Otto M., and Gao Q. (2011) The eukaryotic-type serine/threonine protein kinase stk is required for biofilm formation and virulence in *Staphylococcus epidermidis*. PLoS ONE 6, e25380 21966513 10.1371/journal.pone.0025380 PMC3179523

[23] Pensinger D. A., Boldon K. M., Chen G. Y., Vincent W. J. B., Sherman K., Xiong M., Schaenzer A. J., and Forster E. R. (2016) The *Listeria monocytogenes* PASTA kinase PrkA and its substrate YvcK are required for cell wall homeostasis, metabolism, and virulence. PLoS Pathog. 10.1371/journal.ppat.1006001 PMC5091766 27806131

[24] Tamber S., Schwartzman J., and Cheung A. L. (2010) Role of PknB kinase in antibiotic resistance and virulence in community-acquired methicillin-resistant *Staphylococcus aureus* strain USA300. Infect. Immun. 78, 3637–3646 20547748 10.1128/IAI.00296-10 PMC2916262

[25] Kristich C. J., Wells C. L., and Dunny G. M. (2007) A eukaryotic-type Ser/Thr kinase in *Enterococcus faecalis* mediates antimicrobial resistance and intestinal persistence. Proc. Natl. Acad. Sci. U.S.A. 104, 3508–3513 17360674 10.1073/pnas.0608742104 PMC1805595

[26] Chawla Y., Upadhyay S., Khan S., Nagarajan S. N., Forti F., and Nandicoori V. K. (2014) Protein kinase B (PknB) of *Mycobacterium tuberculosis* is essential for growth of the pathogen *in vitro* as well as for survival within the host. J. Biol. Chem. 289, 13858–13875 24706757 10.1074/jbc.M114.563536 PMC4022859

[27] Canova M. J., Baronian G., Brelle S., Cohen-Gonsaud M., Bischoff M., and Molle V. (2014) A novel mode of regulation of the *Staphylococcus aureus* vancomycin-resistance-associated response regulator VraR mediated by Stk1 protein phosphorylation. Biochem. Biophys. Res. Commun. 447, 165–171 24704444 10.1016/j.bbrc.2014.03.128

[28] Débarbouillé M., Dramsi S., Dussurget O., Nahori M. A., Vaganay E., Jouvion G., Cozzone A., Msadek T., and Duclos B. (2009) Characterization of a serine/threonine kinase involved in virulence of *Staphylococcus aureus*. J. Bacteriol. 191, 4070–4081 19395491 10.1128/JB.01813-08 PMC2698471

[29] Didier J. P., Cozzone A. J., and Duclos B. (2010) Phosphorylation of the virulence regulator SarA modulates its ability to bind DNA in *Staphylococcus aureus*. FEMS Microbiol. Lett. 306, 30–36 20337713 10.1111/j.1574-6968.2010.01930.x

[30] Sun F., Ding Y., Ji Q., Liang Z., Deng X., Wong C. C., Yi C., Zhang L., Xie S., Alvarez S., Hicks L. M., Luo C., Jiang H., Lan L., and He C. (2012) Protein cysteine phosphorylation of SarA/MgrA family transcriptional regulators mediates bacterial virulence and antibiotic resistance. Proc. Natl. Acad. Sci. U.S.A. 109, 15461–15466 22927394 10.1073/pnas.1205952109 PMC3458358

[31] Burnside K., Lembo A., de los Reyes M., Iliuk A., BinhTran N. T., Connelly J. E., Lin W. J., Schmidt B. Z., Richardson A. R., Fang F. C., Tao W. A., and Rajagopal L. (2010) Regulation of hemolysin expression and virulence of *Staphylococcus aureus* by a serine/threonine kinase and phosphatase. PLoS ONE 10.1371/journal.pone.0011071 PMC2884019 20552019

[32] Cheung A., and Duclos B. (2012) Stp1 and Stk1: the yin and yang of vancomycin sensitivity and virulence in vancomycin-intermediate *Staphylococcus aureus* strains. J. Infect. Dis. 205, 1625–1627 22492848 10.1093/infdis/jis255

[33] Fernandez P., Saint-Joanis B., Barilone N., Jackson M., Gicquel B., Cole S. T., and Alzari P. M. (2006) The Ser/Thr protein kinase PknB is essential for sustaining mycobacterial growth. J. Bacteriol. 188, 7778–7784 16980473 10.1128/JB.00963-06 PMC1636329

[34] Pensinger D. A., Aliota M. T., Schaenzer A. J., Boldon K. M., Ansari I. U., Vincent W. J., Knight B., Reniere M. L., Striker R., and Sauer J. D. (2014) Selective pharmacologic inhibition of a PASTA kinase increases *Listeria monocytogenes* susceptibility to β-lactam antibiotics. Antimicrob. Agents Chemother. 58, 4486–4494 24867981 10.1128/AAC.02396-14 PMC4135996

[35] Rüegg U. T., and Burgess G. M. (1989) Staurosporine, K-252 and UCN-01: potent but nonspecific inhibitors of protein kinases. Trends Pharmacol. Sci. 10, 218–220 2672462 10.1016/0165-6147(89)90263-0

[36] Liao J. J. (2007) Molecular recognition of protein kinase binding pockets for design of potent and selective kinase inhibitors. J. Med. Chem. 50, 409–424 17266192 10.1021/jm0608107

[37] Drewry D. H., Willson T. M., and Zuercher W. J. (2014) Seeding collaborations to advance kinase science with the GSK Published Kinase Inhibitor Set (PKIS). Curr. Top. Med. Chem. 14, 340–342 24283969 10.2174/1568026613666131127160819 PMC4435035

[38] Elkins J. M., Fedele V., Szklarz M., Abdul Azeez K. R., Salah E., Mikolajczyk J., Romanov S., Sepetov N., Huang X. P., Roth B. L., Al Haj Zen A., Fourches D., Muratov E., Tropsha A., Morris J., et al (2016) Comprehensive characterization of the Published Kinase Inhibitor Set. Nat. Biotechnol. 34, 95–103 26501955 10.1038/nbt.3374

[39] Donat S., Streker K., Schirmeister T., Rakette S., Stehle T., Liebeke M., Lalk M., and Ohlsen K. (2009) Transcriptome and functional analysis of the eukaryotic-type serine/threonine kinase PknB in *Staphylococcus aureus*. J. Bacteriol. 191, 4056–4069 19376851 10.1128/JB.00117-09 PMC2698490

[40] Heerding D. A., Rhodes N., Leber J. D., Clark T. J., Keenan R. M., Lafrance L. V., Li M., Safonov I. G., Takata D. T., Venslavsky J. W., Yamashita D. S., Choudhry A. E., Copeland R. A., et al (2008) Identification of 4-(2-(4-amino-1,2,5-oxadiazol-3-yl)-1-ethyl-7{[(3S)-3-piperidinylmethyl]oxy}-1H-imidazo[4,5-c]pyridin-4-yl)-2-methyl-3-butyn-2-ol (GSK690693), a novel inhibitor of AKT kinase. J. Med. Chem. 51, 5663–5679 18800763 10.1021/jm8004527

[41] Boudreau M. A., Fishovitz J., Llarrull L. I., Xiao Q., and Mobashery S. (2015) Phosphorylation of BlaR1 in manifestation of antibiotic resistance in methicillin-resistant *Staphylococcus aureus* and its abrogation by small molecules. ACS Infect. Dis. 1, 454–459 27623311 10.1021/acsinfecdis.5b00086 PMC5022795

[42] Vornhagen J., Burnside K., Whidbey C., Berry J., Qin X., and Rajagopal L. (2015) Kinase inhibitors that increase the sensitivity of methicillin resistant *Staphylococcus aureus* to β-lactam antibiotics. Pathogens 4, 708–721 26506394 10.3390/pathogens4040708 PMC4693160

[43] Kant S., Asthana S., Missiakas D., and Pancholi V. (2017) A novel STK1-targeted small-molecule as an “antibiotic resistance breaker” against aureus. Sci. Rep. 10.1038/s41598-017-05314-z PMC5505960 28698584

[44] Tsatsanis C., and Spandidos D. A. (2000) The role of oncogenic kinases in human cancer (review). Int. J. Mol. Med. 5, 583–590 10812005 10.3892/ijmm.5.6.583

[45] Bellacosa A., Kumar C. C., Di Cristofano A., and Testa J. R. (2005) Activation of AKT kinases in cancer: implications for therapeutic targeting. Adv. Cancer Res. 94, 29–86 16095999 10.1016/S0065-230X(05)94002-5

[46] Wu P., Nielsen T. E., and Clausen M. H. (2015) FDA-approved small-molecule kinase inhibitors. Trends Pharmacol. Sci. 36, 422–439 25975227 10.1016/j.tips.2015.04.005

[47] Lougheed K. E., Osborne S. A., Saxty B., Whalley D., Chapman T., Bouloc N., Chugh J., Nott T. J., Patel D., Spivey V. L., Kettleborough C. A., Bryans J. S., Taylor D. L., Smerdon S. J., and Buxton R. S. (2011) Effective inhibitors of the essential kinase PknB and their potential as anti-mycobacterial agents. Tuberculosis 91, 277–286 21482481 10.1016/j.tube.2011.03.005 PMC3158675

[48] Chapman T. M., Bouloc N., Buxton R. S., Chugh J., Lougheed K. E., Osborne S. A., Saxty B., Smerdon S. J., Taylor D. L., and Whalley D. (2012) Substituted aminopyrimidine protein kinase B (PknB) inhibitors show activity against *Mycobacterium tuberculosis*. Bioorg. Med. Chem. Lett. 22, 3349–3353 22469702 10.1016/j.bmcl.2012.02.107 PMC3368261

[49] Morris G. M., Goodsell D. S., Halliday R. S., Huey R., Hart W. E., Belew R. K., and Olson A. J. (1998) Automated docking using a Lamarckian genetic algorithm and an empirical binding free energy function. J. Comput. Chem. 19, 1639–1662

[50] Francino M. P. (2015) Antibiotics and the human gut microbiome: dysbioses and accumulation of resistances. Front. Microbiol. 6, 1543 26793178 10.3389/fmicb.2015.01543 PMC4709861

[51] Langdon A., Crook N., and Dantas G. (2016) The effects of antibiotics on the microbiome throughout development and alternative approaches for therapeutic modulation. Genome Med. 8, 39 27074706 10.1186/s13073-016-0294-z PMC4831151

[52] Ortiz-Lombardía M., Pompeo F., Boitel B., and Alzari P. M. (2003) Crystal structure of the catalytic domain of the PknB serine/threonine kinase from *Mycobacterium tuberculosis*. J. Biol. Chem. 278, 13094–13100 12551895 10.1074/jbc.M300660200

[53] Xu J., Wang J. X., Zhou J. M., Xu C. L., Huang B., Xing Y., Wang B., Luo R., Wang Y. C., You X. F., Lu Y., and Yu L. Y. (2017) A novel protein kinase inhibitor IMB-YH-8 with anti-tuberculosis activity. Sci. Rep. 7, 5093 28698545 10.1038/s41598-017-04108-7 PMC5506005

[54] Hall C. L., Tschannen M., Worthey E. A., and Kristich C. J. (2013) IreB, a Ser/Thr kinase substrate, influences antimicrobial resistance in *Enterococcus faecalis*. Antimicrob. Agents Chemother. 57, 6179–6186 24080657 10.1128/AAC.01472-13 PMC3837872

[55] Åberg E., Lund B., Pflug A., Gani O. A., Rothweiler U., de Oliveira T. M., and Engh R. A. (2012) Structural origins of AGC protein kinase inhibitor selectivities: PKA as a drug discovery tool. Biol. Chem. 393, 1121–1129 23092797 10.1515/hsz-2012-0248

[56] Norman R. A., Toader D., and Ferguson A. D. (2012) Structural approaches to obtain kinase selectivity. Trends Pharmacol. Sci. 33, 273–278 22503441 10.1016/j.tips.2012.03.005

[57] Davis M. I., Hunt J. P., Herrgard S., Ciceri P., Wodicka L. M., Pallares G., Hocker M., Treiber D. K., and Zarrinkar P. P. (2011) Comprehensive analysis of kinase inhibitor selectivity. Nat. Biotechnol. 29, 1046–1051 22037378 10.1038/nbt.1990

[58] Hancock I. C., Wiseman G., and Baddiley J. (1976) Biosynthesis of the unit that links teichoic acid to the bacterial wall: inhibition by tunicamycin. FEBS Lett. 69, 75–80 825388 10.1016/0014-5793(76)80657-6

[59] Brandish P. E., Kimura K. I., Inukai M., Southgate R., Lonsdale J. T., and Bugg T. D. (1996) Modes of action of tunicamycin, liposidomycin B, and mureidomycin A: inhibition of phospho-*N*-acetylmuramyl-pentapeptide translocase from *Escherichia coli*. Antimicrob. Agents Chemother. 40, 1640–1644 8807054 10.1128/aac.40.7.1640 PMC163387

[60] Fiuza M., Canova M. J., Patin D., Letek M., Zanella-Cléon I., Becchi M., Mateos L. M., Mengin-Lecreulx D., Molle V., and Gil J. A. (2008) The MurC ligase essential for peptidoglycan biosynthesis is regulated by the serine/threonine protein kinase PknA in *Corynebacterium glutamicum*. J. Biol. Chem. 283, 36553–36563 18974047 10.1074/jbc.M807175200 PMC2662310

[61] DeLano W. L. (2012) The PyMOL Molecular Graphics System, version 1.5.0.1, Schroedinger, LLC, New York

[62] Jani C., Eoh H., Lee J. J., Hamasha K., Sahana M. B., Han J. S., Nyayapathy S., Lee J. Y., Suh J. W., Lee S. H., Rehse S. J., Crick D. C., and Kang C. M. (2010) Regulation of polar peptidoglycan biosynthesis by Wag31 phosphorylation in mycobacteria. BMC Microbiol. 10, 327 21190553 10.1186/1471-2180-10-327 PMC3019181

[63] Bose J. L., Fey P. D., and Bayles K. W. (2013) Genetic tools to enhance the study of gene function and regulation in *Staphylococcus aureus*. Appl. Environ. Microbiol. 79, 2218–2224 23354696 10.1128/AEM.00136-13 PMC3623228

[64] Kelley L. A., Mezulis S., Yates C. M., Wass M. N., and Sternberg M. J. (2015) The Phyre2 web portal for protein modelling, prediction, and analysis. Nat. Protoc. 10, 845–858 25950237 10.1038/nprot.2015.053 PMC5298202

[65] Tripos (2013) SYBYL-X, verison 2.1.1, Certara USA, Inc., Princeton, NJ

[66] Irwin J. J., Sterling T., Mysinger M. M., Bolstad E. S., and Coleman R. G. (2012) ZINC: a free tool to discover chemistry for biology. J. Chem. Inf. Model 52, 1757–1768 22587354 10.1021/ci3001277 PMC3402020

