# Supplementary material for: A screen for kinase inhibitors identifies antimicrobial imidazopyridine aminofurazans as specific inhibitors of the Listeria monocytogenes PASTA kinase PrkA
Source: J Biol Chem. 2017 Aug 16;292(41):17037–45. doi: 10.1074/jbc.M117.808600 (PMC5641865; doi:10.1074/jbc.M117.808600)
Supplement: Supplemental Data [file supp_292_41_17037__index.html]

A screen for kinase inhibitors identifies antimicrobial imidazopyridine aminofurazans as specific inhibitors of the Listeria monocytogenes PASTA kinase PrkA — A screen for kinase inhibitors identifies antimicrobial imidazopyridine aminofurazans as specific inhibitors of the Listeria monocytogenes PASTA kinase PrkA — Imidazopyridine aminofurazans as PASTA kinase inhibitors — Supplemental Data 

# A screen for kinase inhibitors identifies antimicrobial imidazopyridine aminofurazans as specific inhibitors of the *Listeria monocytogenes* PASTA kinase PrkA

## Supplemental Data

- Kinase Inhibitor Library Screen (.xlsx, 68 KB) - Supplemental data of the kinase inhibitor library screen
- Supplemental Tables (.pdf, 242 KB) - Supplemental tables of strains, plasmids, and primers used
- Supplemental Data (.pdf, 662 KB) - Supplemental Figures
