# Supplementary material for: A screen for kinase inhibitors identifies antimicrobial imidazopyridine aminofurazans as specific inhibitors of the Listeria monocytogenes PASTA kinase PrkA
Source: J Biol Chem. 2017 Aug 16;292(41):17037–45. doi: 10.1074/jbc.M117.808600 (PMC5641865; doi:10.1074/jbc.M117.808600)
Supplement: Supplemental Data [file 10.1074_M117.808600_jbc.M117.808600-2.pdf]

Strain	Description	Reference
E. coli		
XL1-Blue	Competent strain of *E. coli* for routine sub-cloning	Agilent Genomics
Rosetta™ (DE3)	BL21 derivative for enhanced expression of proteins with codons rarely found in *E. coli*; Cam^R	EMD Millipore
JDS1509	Rosetta™ (DE3) carrying pGEX-2T-PrkA; Cam^R, Amp^R	(1)
JDS299	Rosetta™ (DE3) carrying pGEX-2T-Stk1; Cam^R, Amp^R	(1)
JDS951	XL1-Blue carrying pGEX-2T-Stk1 F150T; Amp^R	this work
JDS999	Rosetta™ (DE3) carrying pGEX-2T-Stk1 F150T; Cam^R, Amp^R	this work
JDS937	XL1-Blue carrying pGEX-2T-PrkA T151F; Amp^R	this work
JDS385	Rosetta™ (DE3) carrying pGEX-2T-PrkA T151F; Cam^R, Amp^R	this work
JDS1350	XL1-Blue carrying pGEX-2T-Stk1 S62A/M73V/L85I; Amp^R	this work
JDS1362	Rosetta™ (DE3) carrying pGEX-2T-Stk1 S62A/M73V/L85I; Cam^R, Amp^R	this work
L. monocytogenes		
10430s	Streptomycin-resistant derivative of strain 10403	(2)
S. aureus		
RN4220	Heavily mutagenized shuttle strain that accepts foreign DNA	(3)
LAC	Community-acquired USA300 MRSA strain isolated from LA County	(4)
JDS827	LAC Δ*stk1*	this work

Plasmid	Description	Reference
pGEX-2T	Commercial plasmid for expressing fusion proteins with thrombin-cleavable N-terminal GST tags; Amp^R	GE Healthcare Life Sciences
pJB38	Allelic-exchange plasmid for *S. aureus*; Amp^R (*E. coli*), Cam^R (*S. aureus*)	(5)
pGEX-2T-Stk1	pGEX-2T plasmid for expression of the Stk1 kinase domain (residues 1-348) from *S. aureus*; Amp^R	(1)
pGEX-2T-PrkA	pGEX-2T plasmid for expression of the PrkA kinase domain (1-338) from *L. monocytogenes*; Amp^R	(1)
pGEX-2T-Stk1 F150T	pGEX-2T plasmid for expression of the Stk1 kinase domain with F150T mutation; Amp^R	this work
pGEX-2T-PrkA T151F	pGEX-2T plasmid for expression of the PrkA kinase domain with T151F mutation; Amp^R	this work
pGEX-2T-Stk1 S62A/M73V/L85I	pGEX-2T plasmid for expression of the Stk1 kinase domain with S62A/M73V/L85I triple mutation; Amp^R	this work
pJB38-Δstk1	pJB38 containing fusion of 1 kb upstream and downstream of the *stk1* gene for allelic exchange; Amp^R (*E. coli*), Cam^R (*S. aureus*)	this work

Primer	Sequence (5’ → 3’) (Restriction Sites Lower Case)	Reference
JDS88 (Stk1 KO A)	TATTATgagctcGGTCTGTTTGGTGGTGTGAATGAC	this work
BK34 (Stk1 KO B)	ATACATCATCATAGCTGACTTCTTTTTCAGCTTACCTATCATACTTTATCACCTTCAATAGC	this work
BK35 (Stk1 KO C)	GCTATTGAAGGTGATAAAGTATGATAGGTAAGCTGAAAAAGAAGTCAGCTATGATGATGTAT	this work
JDS89 (Stk1 KO D)	ATTATAcccgggCAATTCCTAAACTTACATGTTTCACCATATCG	this work

SI REFENCES

1. Pensinger, D. a., Aliota, M. T., Schaenzer, A. J., Boldon, K. M., Ansari, I. U. H., Vincent, W. J. B., Knight, B., Reniere, M. L., Striker, R., and Sauer, J. D. (2014) Selective pharmacologic inhibition of a PASTA kinase increases Listeria monocytogenes susceptibility to β-lactam antibiotics. *Antimicrob. Agents Chemother.* **58**, 4486–4494
2. Bishop, D. K., and Hinrichs, D. J. (1987) Adoptive transfer of immunity to Listeria monocytogenes. The influence of in vitro stimulation on lymphocyte subset requirements. *J Immunol*. **139**, 2005–2009
3. Kreiswirth, B. N., Löfdahl, S., Betley, M. J., O'Reilly, M., Schlievert, P. M., Bergdoll, M. S., and Novick, R. P. (1983) The toxic shock syndrome exotoxin structural gene is not detectably transmitted by a prophage. *Nature*. **305**, 709–12
4. Diep, B. A., Gill, S. R., Chang, R. F., Phan, T. H. Van, Chen, J. H., Davidson, M. G., Lin, F., Lin, J., Carleton, H. A., Mongodin, E. F., Sensabaugh, G. F., and Perdreau-Remington, F. (2006) Complete genome sequence of USA300, an epidemic clone of community-acquired meticillin-resistant Staphylococcus aureus. *Lancet*. **367**, 731–739
5. Bose, J. L., Fey, P. D., and Bayles, K. W. (2013) Genetic tools to enhance the study of gene function and regulation in Staphylococcus aureus. *Appl. Environ. Microbiol.* **79**, 2218–2224
