# Supplementary material for: A screen for kinase inhibitors identifies antimicrobial imidazopyridine aminofurazans as specific inhibitors of the Listeria monocytogenes PASTA kinase PrkA
Source: J Biol Chem. 2017 Aug 16;292(41):17037–45. doi: 10.1074/jbc.M117.808600 (PMC5641865; doi:10.1074/jbc.M117.808600)
Supplement: Supplemental Data [file 10.1074_M117.808600_jbc.M117.808600-3.pdf]

A Screen of Human Kinase Inhibitors Identifies Microbiologically Active Imidazopyridine Aminofurazans as Selective Inhibitors of the PASTA Kinase PrkA

Adam J. Shaenzer[1,2], Nathan Wlodarchak[1,2], David H. Drewry[3], William J. Zuercher[3], Warren E. Rose[4], Rob Striker[1,2,5], John-Demian Sauer[1#]

[1]Department of Medical Microbiology and Immunology and [2]Department of Medicine, University of Wisconsin-Madison, Madison WI 53706 [3]SGC-UNC, UNC Eshelman School of Pharmacy, University of North Carolina at Chapel Hill, Chapel Hill NC 27599 [4]School of Pharmacy, University of Wisconsin-Madison, Madison WI 53705 [5]W. S. Middleton Memorial Veteran's Hospital, Department of Molecular and Cell Biology

List of Materials

PDF file of supplemental figures (S1-S5)

Excel spreadsheet of kinase inhibitor library screen data ("Kinase Inhibitor Library Screen Supplementary Data")

PDF file of supplemental tables for strains, plasmids, and primers used in this study

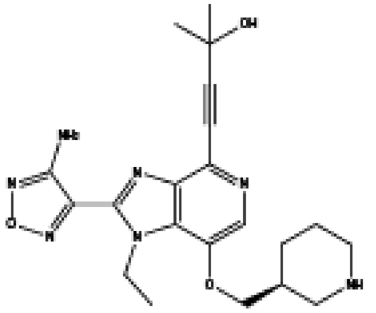

GSK690693

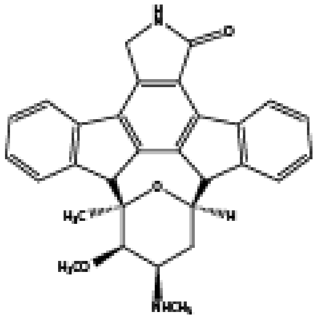

Staurosporine

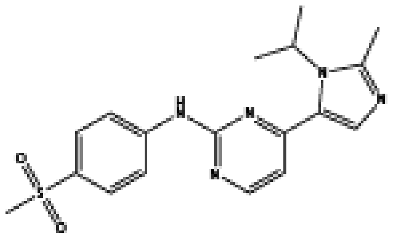

AZD5438

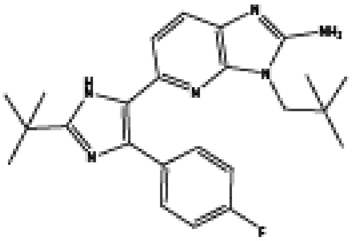

LY2228820

Figure S1: Validated hits from the kinase inhibitor library screen. Compounds displayed were identified as compounds that passed the primary screen (sensitize *L. monocytogenes* to a β-lactam) then passed secondary screens for dose dependence, β-lactam dependence, and PrkA dependence.

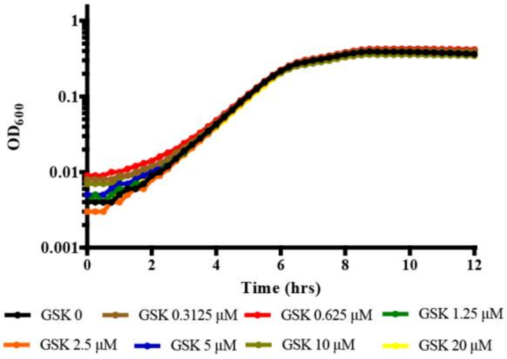

Figure S2: GSK690693 is nontoxic to *L. monocytogenes.* WT *L. monocytogenes* was grown for 12 hours in the presence of varying concentrations of GSK690693. Curves are representative of 3 independent trials.

A

Compound	IC_{50} (µM)	Ceftriaxone MIC (µg/mL)	Oxacillin MIC (µg/mL)
DMSO	*N/A*	32	16
GSK690693	> 40	32	16

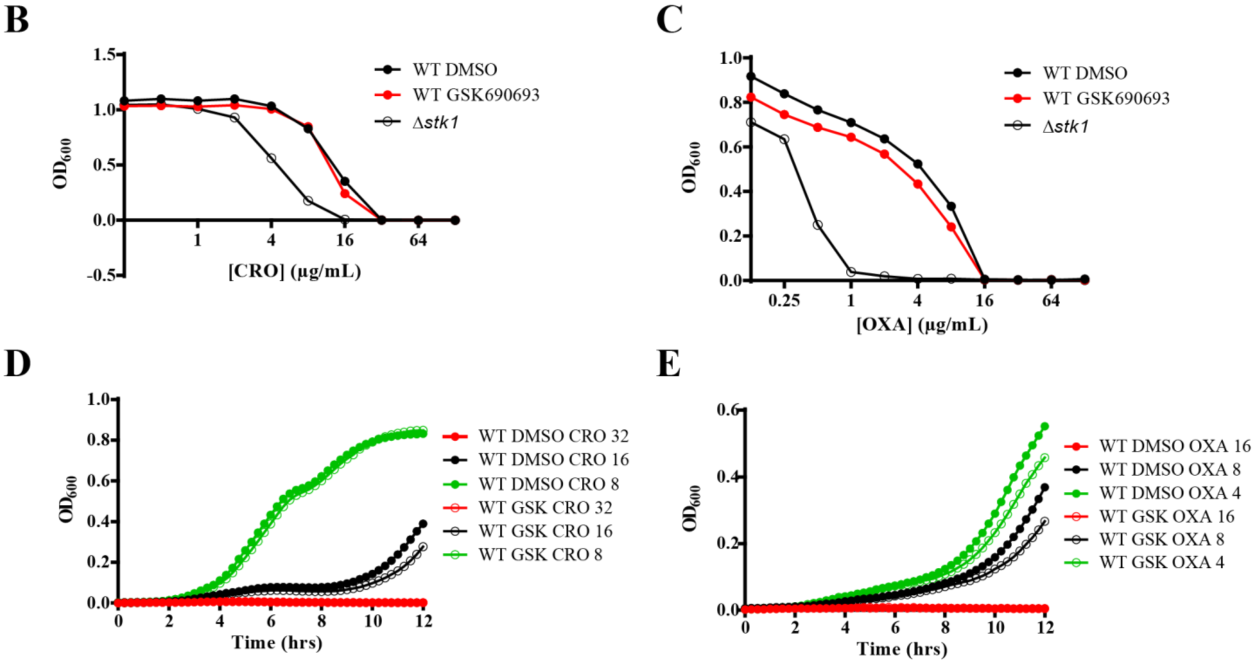

Figure S3: GSK690693 is unable to potentiate β-lactam activity against *S. aureus*. A) Summary of biochemical and microbiology data for GSK690693 against *S. aureus* Stk1. IC_{50} values were determined by Kinase-Glo® assay. N/A: Not applicable. **B-C)** Dose-response curves of WT *S. aureus* LAC and Δ*stk1* growth vs **B)** ceftriaxone and **C)** oxacillin in the presence and absence of 20 µM GSK690693. Curves are representative of 3 independent trials. **D-E)** Growth curves for data presented in **B)** and **C)**, respectively.

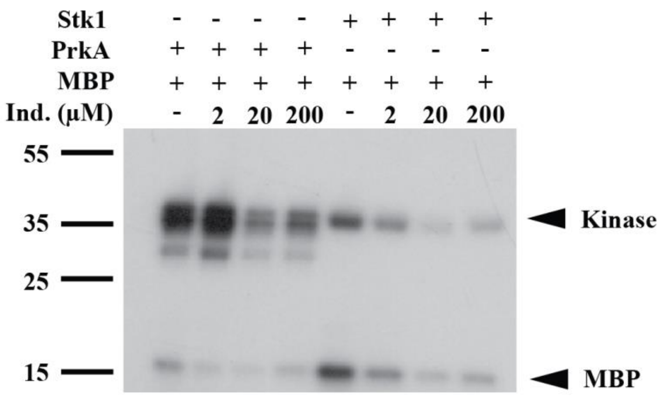

Figure S4: Indirubin-3’-monoxime shows selectivity for Stk1 over PrkA. Autoradiography blots of purified PrkA kinase domain from *L. monocytogenes* or purified Stk1 kinase domain from *S. aureus* and the non-specific phosphoacceptor substrate myelin basic protein (MBP) in the presence or absence of indirubin-3’-monoxime.

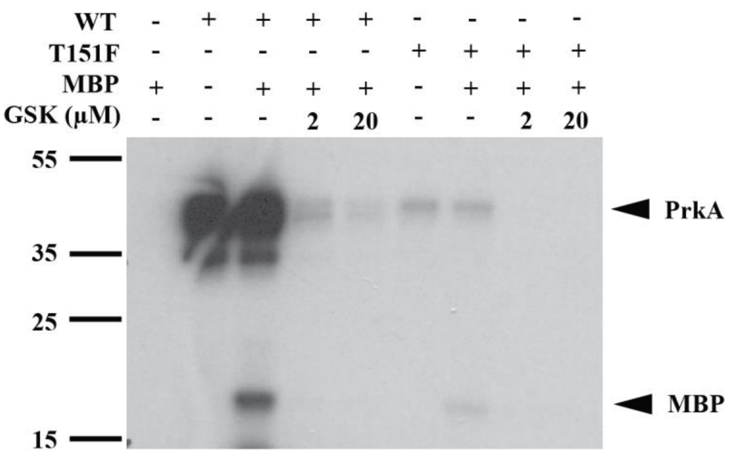

Figure S5: PrkA T151F mutant has reduced activity. Autoradiography blot of purified WT PrkA kinase domain (WT), T151F mutant (T151F), and MBP in the presence or absence of GSK690693. Blot is representative of two independent trials.
